# Simultaneous perturbation of the MAPK and the PI3K/mTOR pathways does not lead to increased radiosensitization

**DOI:** 10.1186/s13014-015-0514-5

**Published:** 2015-10-24

**Authors:** Sebastian Kuger, Michael Flentje, Cholpon S. Djuzenova

**Affiliations:** Department of Radiation Oncology, University Hospital of Würzburg, Würzburg, Germany

**Keywords:** AZD6244, NVP-BEZ235, Radiosensitivity, Cell cycle arrest, Apoptosis, Autophagy

## Abstract

**Background:**

The mitogen-activated protein kinases (MAPK) and the phosphatidylinositol-3-kinase (PI3K)/mammalian target of rapamycin (mTOR) pathways are intertwined on various levels and simultaneous inhibition reduces tumorsize and prolonges survival synergistically. Furthermore, inhibiting these pathways radiosensitized cancer cells in various studies. To assess, if phenotypic changes after perturbations of this signaling network depend on the genetic background, we integrated a time series of the signaling data with phenotypic data after simultaneous MAPK/ERK kinase (MEK) and PI3K/mTOR inhibition and ionizing radiation (IR).

**Methods:**

The MEK inhibitor AZD6244 and the dual PI3K/mTOR inhibitor NVP-BEZ235 were tested in glioblastoma and lung carcinoma cells, which differ in their mutational status in the MAPK and the PI3K/mTOR pathways. Effects of AZD6244 and NVP-BEZ235 on the proliferation were assessed using an ATP assay. Drug treatment and IR effects on the signaling network were analyzed in a time-dependent manner along with measurements of phenotypic changes in the colony forming ability, apoptosis, autophagy or cell cycle.

**Results:**

Both inhibitors reduced the tumor cell proliferation in a dose-dependent manner, with NVP-BEZ235 revealing the higher anti-proliferative potential. Our Western blot data indicated that AZD6244 and NVP-BEZ235 perturbed the MAPK and PI3K/mTOR signaling cascades, respectively. Additionally, we confirmed crosstalks and feedback loops in the pathways. As shown by colony forming assay, the AZD6244 moderately radiosensitized cancer cells, whereas NVP-BEZ235 caused a stronger radiosensitization. Combining both drugs did not enhance the NVP-BEZ235-mediated radiosensitization. Both inhibitors caused a cell cycle arrest in the G1-phase, whereas concomitant IR and treatment with the inhibitors resulted in cell line- and drug-specific cell cycle alterations. Furthermore, combining both inhibitors synergistically enhanced a G1-phase arrest in sham-irradiated glioblastoma cells and induced apoptosis and autophagy in both cell lines.

**Conclusion:**

Perturbations of the MEK and the PI3K pathway radiosensitized tumor cells of different origins and the combination of AZD6244 and NVP-BEZ235 yielded cytostatic effects in several tumor entities. However, this is the first study assessing, if the combination of both drugs also results in synergistic effects in terms of radiosensitivity. Our study demonstrates that simultaneous treatment with both pathway inhibitors does not lead to synergistic radiosensitization but causes cell line-specific effects.

**Electronic supplementary material:**

The online version of this article (doi:10.1186/s13014-015-0514-5) contains supplementary material, which is available to authorized users.

## Background

Standard therapy for solid tumors traditionally consists of different approaches, including surgical resection, hormone therapy, systemic chemotherapy and radiotherapy. However, within the last years combining the traditional approaches with molecular targeted therapies, using monoclonal antibodies and small molecule inhibitors, has become more and more important [71]. Prime targets for this strategy for tumor control are oncogenic signaling cascades, such as the januskinase/signal transducers and activators of transcription, the tumor necrosis factor signaling or the mitogen-activated protein kinases (MAPK) pathway. Especially the MAPK signaling pathway has been shown to stimulate proliferation, cell growth, survival and resistance to chemotherapeutics and ionizing radiation IR [7, 10, 12]. In particular the novel ATP non-competitive MEK inhibitor AZD6244 (generic names: Selumetinib, ARRY-142886) demonstrated high specificity and anti-proliferative activity in *in vitro* and *in vivo* models [69]. Various research groups demonstrated, that apart from the cytostatic effects, AZD6244 also sensitized human tumor cell lines of different origins to IR, underlining the potential of the MAPK pathway as a target for radiosensitization [9, 10, 62].

Another important oncogenic signaling cascade for a molecular targeted therapy is the phosphatidylinositol 3-kinase (PI3K)/mammalian target of rapamycin (mTOR) pathway, which is also related to proliferation and therapy resistance and which also has been validated as a target for radiosensitizing approaches in various *in vitro* and *in vivo* studies [8, 19, 32, 40, 58]. Especially the dual PI3K/mTOR inhibitor NVP-BEZ235 revealed a promising radiosensitizing potential in several experiments [20, 21, 37, 38, 49].

Although, first promising results were obtained for signaling cascade inhibitors in cancers depending on mutations of a single signaling pathway, only limited treatment success was observed, when multiple signaling cascades were deregulated [15, 16, 27], indicating a dependency on the individual mutational background. One possible reason for this limited therapy success is the compensatory up regulation of (other) pathways by feedback loops and/or crosstalks after drug treatment. Such compensatory activation has been shown for a number of cell lines of different tumor entities pointing to its involvement in treatment resistance [34, 35, 42]. Apart from this cell specific a priori resistance to various drugs, the perturbation of a signaling pathway can also result in an acquired drug resistance of initially responsive tumor cells, which ultimately leads to treatment failure [31]. One approach to avoid this resistance by the induction of complementary signaling after drug treatment is to combine inhibitors of different pathways in order to achieve synergistic effects by inhibiting the complementary signaling cascades. In fact, it was proven in several *in vitro* and *in vivo* studies, that simultaneous perturbation of the MAPK and the PI3K/mTOR pathways resulted in enhanced effects compared to single pathway inhibition [5, 25, 53, 66].

Especially the MEK inhibitor AZD6244 and the dual PI3K/mTOR inhibitor NVP-BEZ235 demonstrated synergistic effects in several *in vitro* studies investigating various tumor entities [24, 26, 53, 56, 59]. Furthermore, the promising *in vitro* effects of the combined treatment with AZD6244 and NVP-BEZ235 were already validated in several xenografts in vivo studies with cells of different tumor entities, showing significant synergistic effects including increased tumor shrinkage and prolonged median survival after combined treatment [17, 47, 52, 63].

Although there are several publications, validating the synergistic effects of simultaneous treatment with AZD6244 and NVP-BEZ235, to our knowledge there is no study available evaluating if these synergistic effects are enhanced, when the drugs are combined with IR. To assess the effects of simultaneous MEK and PI3K/mTOR inhibition on the MAPK and PI3K/mTOR signaling cascades and to integrate these data with the phenotypic data of the radiation response after simultaneous MEK and PI3K/mTOR inhibition, we treated glioblastoma SNB19 and lung carcinoma A549 cells with AZD6244 and NVP-BEZ235 alone and in combination. The two cell lines differ in their mutational status, as shown in Table 1, which summarizes mutations of known cancer genes in the two cell lines [30]. As illustrated in Table 1, both cell lines have a common mutation in CDK2NA, which codes for the tumor suppressor protein p16. However, the two cell lines differ in their mutational status regarding the oncogenic MAPK and PI3K/mTOR pathways. SNB19 cells are not expressing functional phosphatase and tensin homologue (PTEN), which is a negative regulator of the PI3K/mTOR signaling cascade [70]. A549 lung carcinoma cells in contrast do not have any known mutations in the PI3K/mTOR pathway, but a mutation in the *Kirsten rat sarcoma viral oncogene homolog* (*KRAS*) gene, which occurs in about 30 % of non small cell lung cancers [6]. These mutations result in a constitutive active form of the protein [1], ultimately leading to activation of the MAPK signaling cascade.

**Table 1:** Mutations of known cancer genes in the glioblastoma SNB19 and the lung carcinoma A549 cell lines [30]

*Cell line*	*Gene*	*Variant*	*Effect*
SNB19	CDKN2A	Hom c.1_471 del 471, p.?	LOM
	PTEN	Hom c.723_724insTT p.E242fsX15	LOM
	TP53	Hom c.818G > A, p.R273H	LOM
A549	CDKN2A	Hom c.1_471 del 471, p.?	LOM
	KRAS	Hom c.34G > A, p.G12S	LOM
	STK11	Hom c.109C > T, p.Q37X	LOM

Abbreviations: *Hom* homozygous, *del* deletion, *LOM* likely oncogenic mutation

After determining the effects of AZD6244 and NVP-BEZ235 on the cellular proliferation rates and the expression levels of several key proteins of the MAPK (Raf-1, p-MEK1/2, MEK2, p-Erk1/2 and Erk2) and PI3K/mTOR signaling cascades (PI3K p110, PI3K p85, PTEN, p-Akt, Akt, p-mTOR, mTOR, p-S6, S6 and p-4E-BP1), we assessed the colony forming abilities, the cell cycle phase distributions, the expression levels of cell cycle related proteins (CDK1, CDK4 and p-Rb), the incidence of apoptosis markers (hypodiploid cells and poly (ADP-Ribose) polymerase (PARP) expression levels and cleavage) and the expression levels of autophagy related proteins (LC3-I and LC3-II) dependent on drug treatment and IR.

## Methods

### Cell culture and drug treatment

The human lung cancer cell line A549 and the human glioblastoma cell line SNB19 were obtained from the “Cell Line Services” company (Heidelberg, Germany) and routinely cultured under standard conditions (37 °C, 5 % CO_2_) in Dulbecco’s modified Eagle’s medium supplemented with 10 % FBS, 1 % glutamine and 1 % Penicillin-Streptomycin. For the proliferation assays cells were treated for 24 h with the indicated concentrations of AZD6244 (Selleckchem, Houston, TX, USA) and NVP-BEZ235 (Novartis Institutes for Biomedical Research, Basel, Switzerland) before measurement of the ATP content. For the other experiments of this study cells were treated 16 or 1 h prior to IR with 500 nM AZD6244 or 50 nM NVP-BEZ235, respectively. Drugs were freshly diluted from frozen aliquots stored at −20 °C. Cells treated in parallel with dimethylsulfoxide (DMSO) served as controls.

### Cell viability assay

The proliferation rate was analyzed with the CellTiter-Glo Luminescent Cell Viability Assay (Promega, Madison, WI, USA) according to the manufacturer’s instructions. Serial dilutions of AZD6244 (31.25–4000 nM) or NVP-BEZ235 (3.125-400 nM) were added to exponentially growing cells and the ATP levels were determined 24 h afterwards. Furthermore, experiments with serial dilutions of AZD6244 (31.25–4000 nM) in the presence of 50 nM NVP-BEZ235 and NVP-BEZ235 (3.125–400 nM) in the presence of 500 nM AZD6244 were performed. Experiments were done in triplicates and the mean ATP content data derived from two independent experiments were normalized against DMSO-treated controls to generate dose–response curves. Further analysis of the data was basically performed as described previously [38].

### X-ray IR

IR was performed at room temperature using a 6 MV linear accelerator (Siemens, Concord, USA) at a dose rate of 2 Gy/min. After IR, cells were cultured in standard conditions for the indicated time until harvest.

### Western blot

The preparation of whole cell lysates, separation according the protein size using Western blotting techniques and the detection of protein levels using protein-specific primary and species-specific peroxidase-labeled secondary antibodies were performed according standard protocols as described previously [68]. The antibodies used in this study are specified in Additional file 1. Protein expression levels were quantified using ImageJ (NIH, Bethesda, MD, USA), normalized to β-actin levels and the relative protein expressions of the shown representative biological replicate are denoted by numbers below the corresponding blot (if changes between treatments were observed). For each Western blot experiment three independent biological replicates were performed.

### Colony forming assay

Colony forming assays were performed and data were analyzed with the linear quadratic model as described elsewhere [22]. Briefly, cells were treated with 500 nM AZD6244 and/or 50 nM NVP-BEZ235 16 h or 1 h prior to IR, respectively. Twenty-four hours after IR with graded single doses up to 8 Gy the cells were detached and seeded into 6 well plates containing drug free medium. Cells were then cultivated under standard conditions for two weeks. Colonies were stained with 0.6 % crystal violet and colonies containing more than 50 cells, were scored as survivors. Experiments were done in triplicate and each experiment was repeated at least four times.

### Measurement of cell cycle phase distribution and hypodiploid cells

Cell cycle phase distributions and proportion of hypodiploid cells were assessed as described elsewhere [55]. Briefly, samples were fixed 30 min, 24 and 48 h after IR by adding ice cold ethanol. After permeabilization and treatment with RNase A cells were stained with propidium iodide (PI) and at least 20,000 cells were assessed for their DNA content, using a flow cytometer FACSCanto II (Becton Dickinson, San Jose, CA, USA). For cell cycle analysis cell conglomerates and hypodiploid cells were excluded and deconvolution of DNA histograms was performed using the ModFit LT (Verity Software House, Topsham, ME, USA) software. Cells showing less than 80 % of the fluorescence signal of average G1-phase cells were considered to be hypodiploid.

### Software and statistics

Data are represented as means ± standard deviation (SD) of at least three independent experiments. Unpaired two-sided t-tests were performed and *P* values <0.05 were considered to be statistically significant. For multiple comparisons the Holm-Bonferroni method of alpha error correction was applied. Statistical comparison of colony forming assays was done using the statistical software RStudio 0.96.331 (Free Software Foundation, Boston, MA, USA) along with the package CFAssay (H. Braselmann, Helmholtz Zentrum German Research Center for Environmental Health, Munich, Germany). For statistical comparisons of the cell cycle phase distribution data we tested each cell cycle phase (G1-, S- or G2/M-phase) between the different treatment groups (Control, AZD6244, NVP-BEZ235, and AZD6244 + NVP-BEZ235). Statistical significant differences in at least one of the cell cycle phases between the different treatment groups are indicated in the figure. For reasons of clarity we omitted comparisons between unirradiated and irradiated samples. Further software used in this study was Flowing Software (P. Terho, Turku Centre for Biotechnology, Turku, Finland), ImageJ (NIH, Bethesda, MD, USA) and Origin 8.5 (Microcal, Northampton, MA, USA).

## Results

### AZD6244 and NVP-BEZ235 decrease cell proliferation and perturb oncogenic signaling cascades

**Fig. 1:**
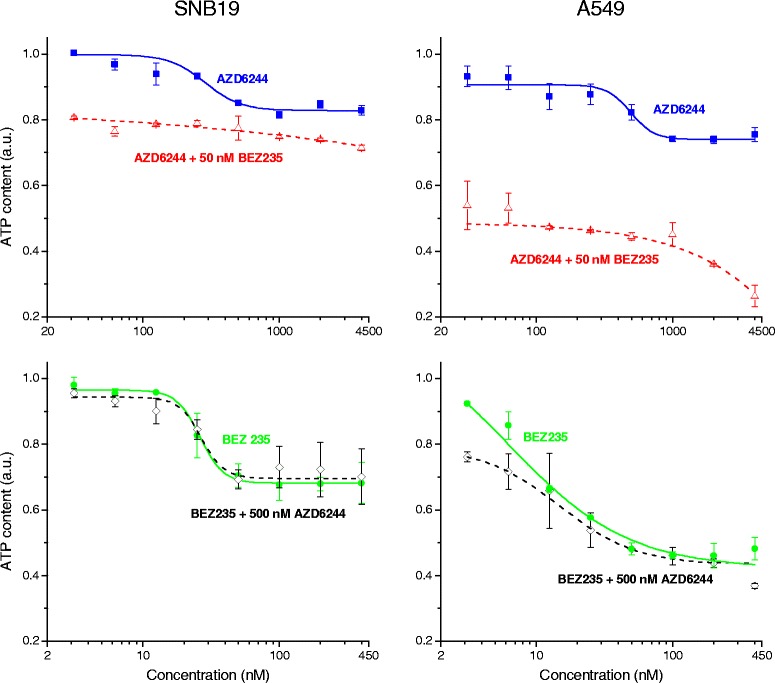
Proliferation assays of SNB19 and A549 after treatment AZD6244 and/or NVP-BEZ235. Effect of 24 h exposure to serial dilutions of AZD6244 (blue squares), AZD6244 with 50 nM NVP-BEZ235 (red triangels), NVP-BEZ235 (green circles) or NVPBEZ235 with 500 nM AZD6244 (black diamonds) on the ATP level in SNB19 or A549 cells, as measured by a standard luciferase assay. The diagram represents the means of two independent experiments, each performed in triplicates, normalized against DMSO-treated controls. Dose–response curves (illustrated in the corresponding color) were generated using the standard four parameter logistic models and error bars indicate SD values

To assess the effects of AZD6244 or NVP-BEZ235 on glioblastoma SNB19 and lung carcinoma A549 cells, we treated both cell lines with serial dilutions of the inhibitors within a concentration range of 31.25-4000 nM for AZD6244 and 3.125-400 nM for NVP-BEZ235 and quantified the cell viability by the CellTiter-Glo Luminescent Cell Viability Assay. ATP content in drug-treated samples was normalized against DMSO treated controls and plotted against the drug concentration. To evaluate the effect of combining both inhibitors, further experiments with serial dilutions of AZD6244 in the presence of 50 nM NVP-BEZ235 or NVP-BEZ235 in the presence of 500 nM AZD6244 were performed. As evident from Fig. 1 an incubation with increasing concentrations of the MEK inhibitor AZD6244 decreased cell proliferation to about 85 % in SNB19 and to about 75 % in A549 cells. An incubation with serial dilutions of the dual PI3K/mTOR inhibitor NVP-BEZ235 also decreased the proliferation rates in both cell lines. In the SNB19 cell line NVP-BEZ235 concentrations higher than 12.5 nM caused a decrease of the relative ATP content to a minimum of approximately 70 %, which was achieved by an incubation with 50 nM or higher concentrations of NVP-BEZ235. Incubation of A549 cells with NVP-BEZ235 already caused a decrease of the relative ATP level at low nanomolar concentrations. A minimum relative ATP level of about 50 % was achieved when the lung carcinoma cells were treated with NVP-BEZ235 concentrations of 50 nM or higher.

To address the question, if there are synergistic effects on the proliferation rate after combing AZD6244 and NVP-BEZ235, we used a combination of both inhibitors with serial dilutions of AZD6244 (31.25–4000 nM) and 50 nM of NVP-BEZ235 or of NVP-BEZ235 (3.125-400 nM) with 500 nM of AZD6244. We chose a concentration of 50 nM for NVP-BEZ235 and 500 nM for AZD6244 since these concentrations are in the range of previously published data regarding radiosensitizing effects of the PI3K/mTOR and MEK inhibitor [9, 20, 21, 37, 51, 62], which we wanted to investigate in combination in this study. As demonstrated in Fig. 1, an incubation of SNB19 cells with increasing AZD6244 concentrations along with 50 nM of NVP-BEZ235 resulted in a relative ATP level of about 70 %, which was also achieved by incubation with 50 nM NVP-BEZ235 alone. In A549 cells incubation with 31.25-500 nM AZD6244 along with 50 nM NVP-BEZ235 also revealed no synergistic effects. However, when the cells were treated with AZD6244 concentrations higher than 1000 nM in combination with 50 nM NVP-BEZ235 a decrease of the relative ATP level was observed. Incubation of SNB19 cells with increasing NVP-BEZ235 concentrations in combination with 500 nM AZD6244 revealed no further effects on the relative ATP content then treatment with the dual PI3K/mTOR inhibitor alone. The treatment of A549 cells with increasing NVP-BEZ235 concentrations along with 500 nM AZD6244 resulted in a decreased relative ATP level at low nanomolar NVP-BEZ235 concentrations compared to treatment with NVP-BEZ235 solely. However, with NVP-BEZ235 concentrations higher than 10 nM this synergistic effect vanished. For subsequent experiments we used drug concentrations of 500 nM for AZD6244 and 50 nM for NVP-BEZ235, which are in line with previously published experiments [10, 38, 43, 69].

**Fig. 2:**
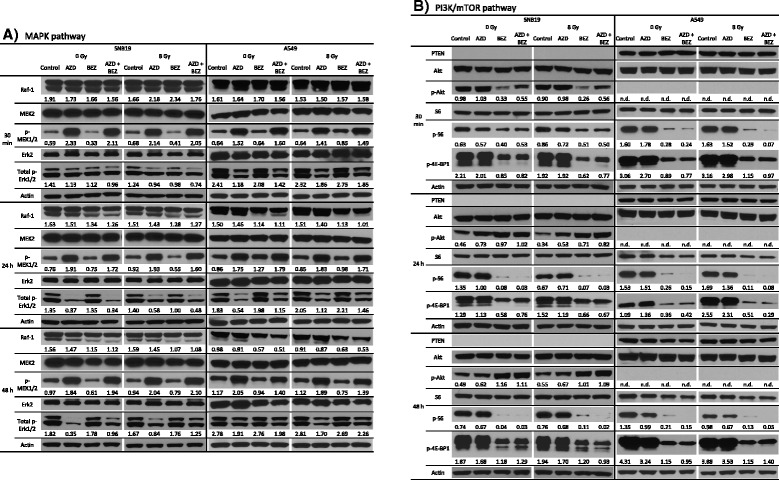
Expression levels of selected proteins of the MAPK/Erk and the PI3K/mTOR pathways in SNB19 and A549 cells. Representative Western blot analysis of expression levels of selected proteins of the MAPK **a** and the PI3K/mTOR pathway **b** in SNB19 and A549 cells. Cells were treated with AZD6244 and/or NVP-BEZ235 before IR with 8 Gy and whole cell lysates were prepared 30 min, 24 h and 48 h after IR. Protein bands were normalized to the β-actin intensity and changes in protein expression are denoted by numbers if applicable. Since the utilized antibody detected p-Erk1 and p-Erk2 the numbers below the blot correspond to the expression levels of total p-Erk1/2

To elucidate the molecular changes for the observed anti-proliferative effects, we assessed the expression and phosphorylation levels of several proteins of the MAPK and the PI3K/mTOR pathway after drug treatment and IR in a time-dependent manner. As shown in Fig. 2a the incubation with the MEK inhibitor AZD6244 resulted in an increased phosphorylation of MEK1/2 and a decreased phosphorylation of Erk1/2 in SNB19 and A549 cells 30 min after IR. These increased p-MEK1/2 and decreased p-Erk1/2 levels were also observed 24 and 48 h after IR in both cell lines (Fig. 2a). The treatment with the dual PI3K/mTOR inhibitor NVP-BEZ235 had only minor effects on the proteins of the MAPK pathway 30 min after IR. However, as demonstrated in Fig. 2b, incubation with NVP-BEZ235 reduced the expression levels of Raf-1 in both cell lines 24 and 48 h after IR.

As illustrated in Fig. 2b, the tumor suppressor protein PTEN was only detectable in *PTEN* wild type A549 cells, whereas it was absent in the SNB19 cell line. This correlated with the expression of p-Akt, which is negatively regulated by PTEN and which was activated in SNB19 cells. As demonstrated in Fig. 2b, which shows the expression of several other proteins of the PI3K/mTOR signaling cascade, incubation with the MEK inhibitor AZD6244 had no effect on the proteins of the PI3K/mTOR pathway. In contrast, the treatment with NVP-BEZ235 resulted in decreased p-Akt levels 30 min after IR in SNB19 cells (Fig. 2b). Interestingly, simultaneous incubation of SNB19 cells with both inhibitors resulted in a diminished decrease of p-Akt compared to the treatment with NVP-BEZ235 alone. However, 24 and 48 h after IR the initially decreased p-Akt levels in NVP-BEZ235-treated SNB19 cells recovered to a higher phosphorylation level of Akt than in the control samples (Fig. 2b). This effect was independent of IR and co-incubation with AZD6244.

Apart from its effect on Akt phosphorylation, incubation of SNB19 cells with NVP-BEZ235 caused a dephosphorylation of 4E-BP1 and a slight dephosphorylation of S6 30 min after IR (Fig. 2b). This NVP-BEZ235 induced dephosphorylation of S6 was fortified 24 and 48 h after IR, thus p-S6 could hardly be detected at these time points. The reduced levels of p-4E-BP1 after incubation with NVP-BEZ235 were measured also 24 and 48 h after IR (Fig. 2b). The inhibition of the PI3K/mTOR pathway by NVP-BEZ235, as indicated by decreased S6 and 4E-BP1 phosphorylation, was observed independently of IR and an incubation with AZD6244. The A549 cell line showed qualitatively similar results, except for Akt phosphorylation, which was not detectable in this cell line. Other proteins of the PI3K/mTOR pathway (PI3K p110, PI3K p85, p-mTOR and mTOR) were also analyzed, but did not reveal any noteworthy changes in protein expression or phosphorylation (data not shown).

**Fig. 3:**
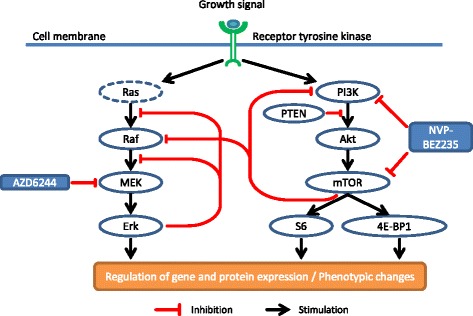
Putative interactions and feedback loops in the MAPK and PI3K/mTOR signaling cascades. Putative signaling diagram of the MAPK and PI3K/mTOR pathways derived from the Western blot analysis shown Fig. 2 and literature data specified in the text. Treatment with the allosteric MEK inhibitor AZD6244 causes desphosphorylation of Erk resulting in phenotypic changes (e.g. changes in the proliferation rate, cell cycle phase distribution and radiosensitivity). However, simultaneously the MAPK pathway is activated through a feedback loop from Erk to the upper part of the signaling cascade resulting in hyperphosphorylation of MEK. Inhibiting the PI3K/mTOR pathway with NVP-BEZ235 caused a dephosphorylation of the transcription and translation regulators S6 and 4E-BP1 also inducing the aforementioned phenotypic changes. However, also for the PI3K/mTOR pathway we observed a feedback loop, as indicated by increased Akt phosphorylation after PI3K and mTOR inhibition in SNB19 cells. Furthermore, we validated a crosstalk between the two signaling cascades since treatment with the dual PI3K/mTOR inhibitor also resulted in decreased Raf-1 expression levels. Stimulation is indicated by black normal arrows, whereas inhibition is indicated by red blunt arrows

A putative signaling diagram, derived from our Western blot and literature data, illustrating the AZD6244 and NVP-BEZ235 induced perturbations in the MAPK and the PI3K/mTOR signaling cascades is depicted in Fig. 3. Inhibition of the MAPK signaling pathway with AZD6244 caused reduced phosphorylation of Erk, but increased the phosphorylation of MEK pointing towards a feedback loop from Erk to the top of the signaling cascade, as depicted in the diagram. Likewise, the perturbation of the PI3K/mTOR pathway with NVP-BEZ235 also caused the induction of a feedback loop, as indicated by the increased phosphorylation of Akt in SNB19 cells. Furthermore, a crosstalk between the PI3K/mTOR and the MAPK pathway was confirmed by Western blotting (Fig. 3). In order to further analyze the phenotypic effects of inhibiting the MAPK and PI3K/mTOR signaling cascades, especially in the light of the radiation response, we measured the colony forming abilities, cell cycle aberrations and the induction of apoptosis and autophagy after treatment with AZD6244 and/or NVP-BEZ235 in irradiated and sham-irradiated cells.

### Effects of AZD6244 and NVP-BEZ235 on the radiosensitivity

**Fig. 4:**
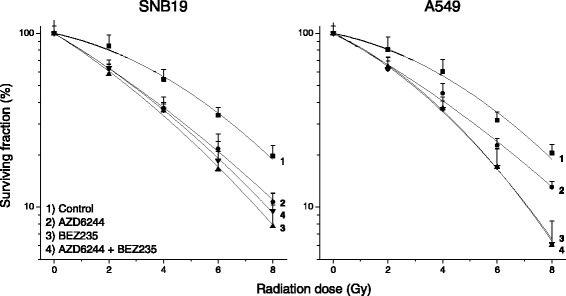
Colony forming abilities of SNB19 and A549 cancer cells as functions of drug and IR exposure. Control (DMSO-treated, empty circles), AZD6244- (filled squares), NVP-BEZ235- (filled triangles) and AZD6244 + NVP-BEZ235-treated cells (filled diamonds) were irradiated with single graded doses up to 8 Gy. Two weeks after IR colonies were fixed and stained using standard protocols. Experiments were performed in triplicates and repeated at least four times. Colonies containing at least 50 cells were scored as survivors

To assess the effects of perturbations in the oncogenic MAPK and PI3K/mTOR signaling cascades on the radiosensitivity, we treated SNB19 and A549 cells with AZD6244, NVP-BEZ235 or a combination of both inhibitors before IR with single doses up to 8 Gy. Figure 4 shows the mean normalized clonogenic survival responses of at least four independent experiments plotted against the radiation dose along with the best fits of the linear quadratic model to the data. The mean plating efficiencies and radiosensitivity parameters derived from the best fits of the linear quadratic models for both cell lines are summarized in Additional file 2.

As illustrated in Fig. 4 and Additional file 2, AZD6244 caused a slight decrease in both, the surviving fraction at 2 Gy (SF2) and the dose yielding 10 % survival (D10), indicating a moderate radiosensitization in both cell lines. The dual PI3K/mTOR inhibitor NVP-BEZ235 sensitized both cell lines to a greater extent, as confirmed by a steeper decline of the survival curve and higher inhibitory factors for SF2 and D10. In both cell lines combined incubation with both inhibitors did not increase radiosensitization compared to inhibition of the PI3K/mTOR signaling cascade with NVP-BEZ235 solely.

### Cell cycle alterations induced by AZD6244, NVP-BEZ235 and IR

**Fig. 5:**
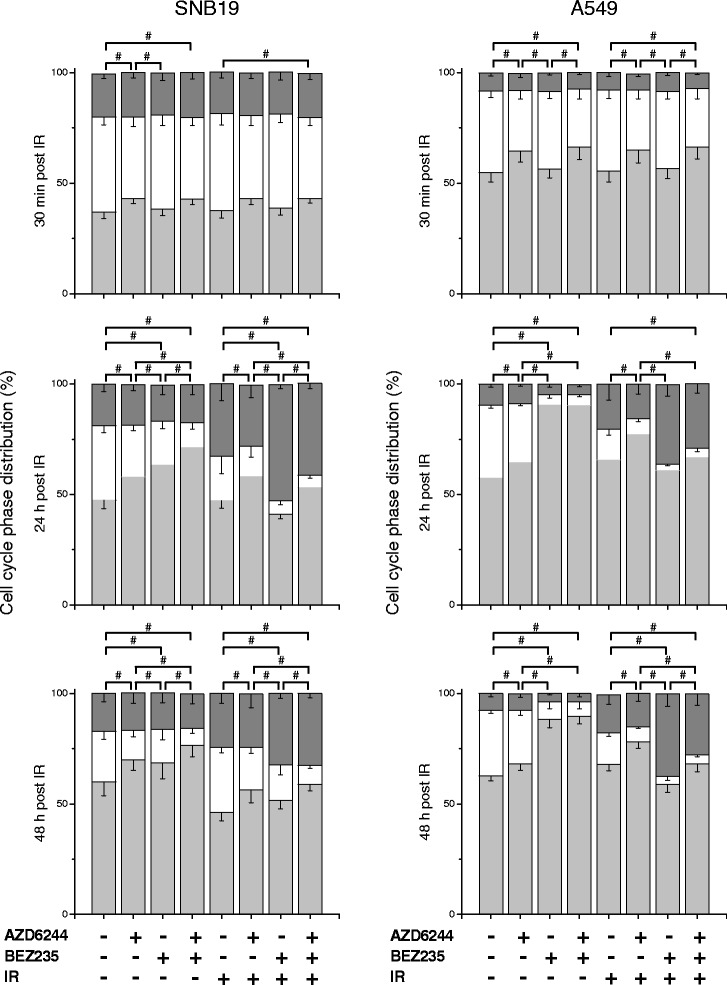
Effects of AZD6244, NVP-BEZ235 and IR on cell cycle phase distributions. Cell cycle phase distribution of SNB19 and A549 tumor cells treated with AZD6244 and/or NVP-BEZ235 before IR with 8 Gy. The cells were fixed 30 min, 24 or 48 h after IR, permeabilized, treated with RNase, stained with PI and analyzed for DNA content by flow cytometry. Data are presented as means (±SD) of G1- (light grey), S- (white) and G2/M-phase (dark grey) cells of at least three independent experiments for each cell line. Statistical significant changes assessed as specified in the Methods section are indicated by # *P* < 0.05

To further explore the phenotypic changes after incubation with AZD6244 and NVP-BEZ235 in SNB19 and A549 cells, we analyzed the cell cycle phase distribution after MEK and PI3K/mTOR inhibition in irradiated and sham-irradiated cells. The summarized data of at least three independent experiments are presented in Fig. 5, whereas representative histograms are presented in Additional file 3. The large percentage of SNB19 and A549 cells in the S and G2/M-phase of the cell cycle indicates, that the cells were in the exponential growth phase at the beginning of the experiments, although cell line specific differences occurred. Incubation for 16 h with AZD6244 prior to IR resulted in a slight elevation of cells in the G1-phase 30 min after IR in both cell lines, whereas neither NVP-BEZ235 nor IR with 8 Gy had an effect on the cell cycle phase distribution at this time point.

As confirmed by the increased fractions of G1-phase cells 24 and 48 h after IR, both inhibitors had an anti-proliferative effect, although an incubation of A549 cells with NVP-BEZ235 resulted in a higher proportion of G1-phase cells than incubation of SNB19 cells. However, the combination of AZD6244 and NVP-BEZ235 synergistically increased the fraction of SNB19 cells, whereas no additional effect were observed in A549 cells, as shown in Fig. 5 and Additional file 3. IR resulted in cell line-specific alterations of the cell cycle, namely in SNB19 cells IR with 8 Gy caused an elevated proportion of G2/M-phase cells, whereas IR of A549 cells resulted in an increase of the G1-phase proportion mainly.

The treatment with AZD6244 prior to IR caused a moderate increase of the G1-phase fraction in addition to the radiation induced cell cycle aberrations in both cell lines. In contrast, perturbation of the PI3K/mTOR signaling cascade with NVP-BEZ235 resulted in elevated G2/M-phase levels in both cell lines up to 48 h after IR. The simultaneous incubation with both inhibitors caused a slight increase of the proportion of cells in the G1-phase (less than in cells treated with AZD6244 alone) and slightly elevated G2/M-phase levels (less than in cells treated with NVP-BEZ235 alone), as shown in Fig. 5 and Additional file 3.

**Fig. 6:**
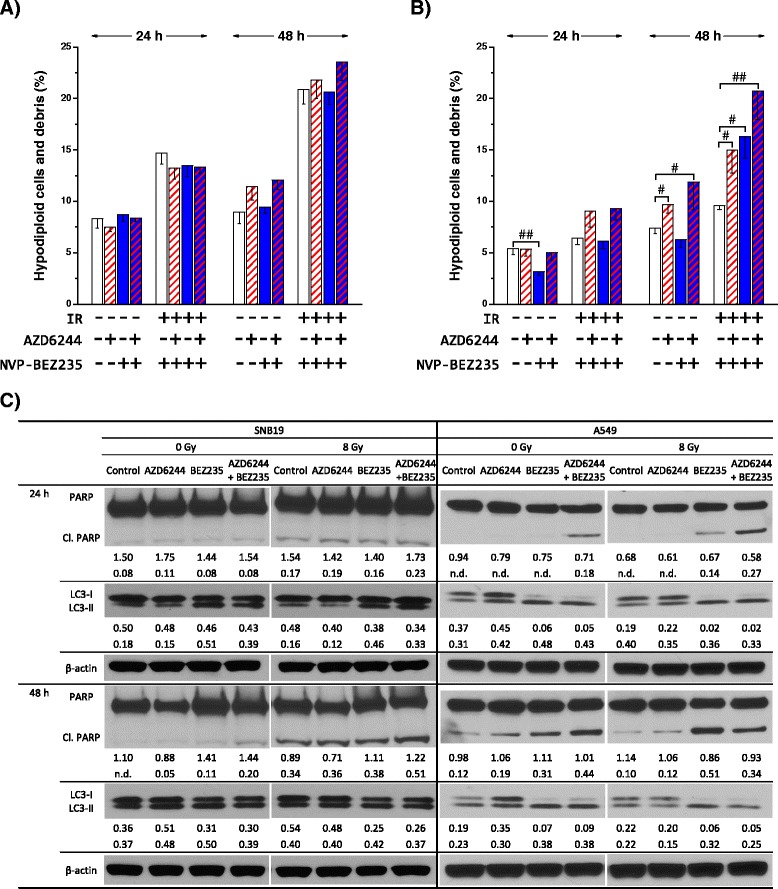
Effects of AZD6244, NVP-BEZ235 and IR on the induction of apoptosis and autophagy. Mean percentage of cells with hypodiploid DNA content and cellular debris in cells treated with AZD6244 (red striped columns), NVP-BEZ235 (blue columns) or a combination of both inhibitors (red and blue striped columns) 24 and 48 h after IR. DMSO treated cells (blank columns) served as controls. Cells were detached with trypsin, fixed, permeabilized, treated with RNase, stained with PI and then analyzed for fluorescence by flow cytometry. The columns display means (±SD) of hypodiploid cells and cellular debris of at least three independent experiments. Statistical significant differences are indicated as follows: # *P* < 0.05; ## *P* <0.01. Statistical significant differences between sham-irradiated and irradiated control samples are not depicted for reasons of clarity. Representative Western blot analysis of expression levels of PARP, cleaved PARP, LC3-I and LC3-II in SNB19 and A549 cells **c**. Cells were treated with AZD6244 and/or NVP-BEZ235 before IR with 8 Gy and whole cell lysates were prepared 24 and 48 h after IR as described previously. Protein bands were normalized to the β-actin intensity and changes in protein expression are denoted by numbers if applicable. The experiment was repeated at least three times

In order to elucidate the molecular basis for the alterations in the cell cycle, observed in SNB19 and A549 cells after IR and/or treatment with the inhibitors, we analyzed the expression of the cell cycle related proteins CDK1, CDK4 and p-Rb. As shown in Fig. 6a, neither each inhibitor alone nor a combination of them had an effect on the expression levels of the tested cell cycle related proteins in both cell lines 30 min after IR. Also the exposure to 8 Gy had no effects on the expression levels of CDK1, CDK4 and p-Rb at this time point.

A perturbation of the MAPK pathway with AZD6244 resulted in decreased expression levels of CDK1 24 and 48 h after IR in SNB19 cells (Fig. 6b and c). This was observed independently of IR or co-incubation with NVP-BEZ235. The phosphorylation of Rb was also reduced in AZD6244 treated SNB19 cells, although a co-incubation with NVP-BEZ235 resulted in a higher reduction than treatment with AZD6244 solely. This synergistic effect of AZD6244 and NVP-BEZ235 correlated with the enhanced G1-phase arrest in SNB19 cells after combined incubation with both inhibitors, as shown in Fig. 5. IR of SNB19 cells had only minor effects on the expression levels of CDK1 and CDK4, whereas an increase of p-Rb was detected 24 h after IR. Again in accordance to the cell cycle phase distribution data, treating SNB19 cells with AZD6244 and IR resulted in decreased levels of the G1/S-phase transition regulator p-Rb.

The A549 cells revealed a different expression pattern of the cell cycle related proteins after IR (Fig. 6b and c). In SNB19 cells incubation with AZD6244 caused a slight reduction of CDK1 expression levels, whereas this was not detected in A549 cells. However, in accordance with the strong G1 arrest induced by NVP-BEZ235 in A549 cells (Fig. 5), the treatment with the dual PI3K/mTOR inhibitor caused a slight decrease of CDK1 and CDK4 expression in A549 cells independent of IR. The phosphorylation level of Rb was slightly reduced by AZD6244 and to a much greater extent by NVP-BEZ235. Simultaneous incubation with both inhibitors only had minor further effects on the p-Rb level in A549 cells, which is in agreement with the cell cycle phase distribution data (as shown in Fig. 5 and Additional file 3). An exposure of the A549 cell line to IR also resulted in a decreased phosphorylation of Rb. This decreased p-Rb levels were further reduced when the A549 cells where incubated with AZD6244. Interestingly the treatment with NVP-BEZ235 further decreased the phosphorylation level of Rb in irradiated A549 cells. As already observed in sham-irradiated A549 cells, also in irradiated A549 cells the NVP-BEZ235 induced reduction of p-Rb was not altered by simultaneous incubation with AZD6244.

### Apoptosis and autophagy induction by AZD6244, NVP-BEZ235 and IR

**Fig. 7:**
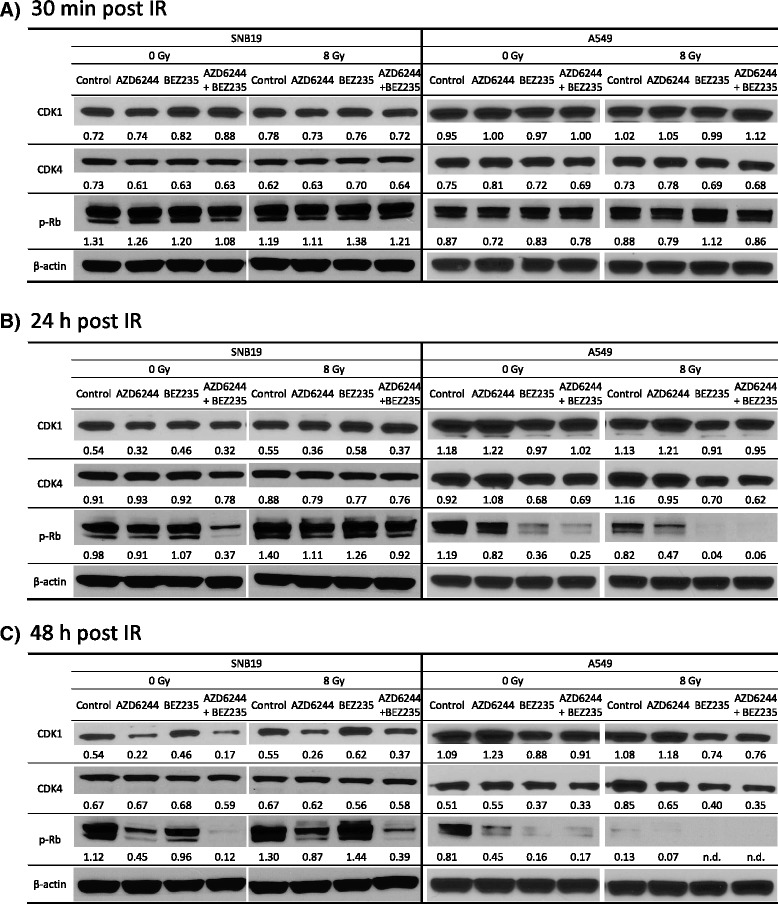
Effects of AZD6244, NVP-BEZ235 and IR on cell cycle related proteins. Representative Western blot analysis of expression levels of selected proteins associated with the cell cycle in SNB19 and A549 cells. Cells were treated with AZD6244 and/or NVP-BEZ235 before IR with 8 Gy and whole cell lysates were prepared 30 min **a**, 24 **b** and 48 h **c** after IR as described previously. Protein bands were normalized to the β-actin intensity and changes in protein expression are denoted by numbers if applicable. The experiment was repeated at least three times

To further elucidate the phenotypic effects of AZD6244 and NVP-BEZ235 in SNB19 and A549 cells, we assessed apoptosis and autophagy induction after incubation with the inhibitors in sham-irradiated and irradiated cells. Figure 7a and b illustrate the mean percentage of cells with hypodiploid DNA content and cellular debris, a marker for late stage apoptosis, summarized from at least three independent experiments. Representative Western blot experiments for the expression of the DNA repair enzyme PARP and its cleavage, as well as expression of the autophagy marker LC3 are represented in Fig. 7c. As shown in Fig. 7a, incubation with each inhibitor alone or in combination did not affect the percentage of hypodiploid cells in sham-irradiated SNB19 cells. In contrast, exposing SNB19 cells to 8 Gy caused an increase of hypodiploid cells, which was not enhanced by any of the two inhibitors or their combination. The data for the hypodiploid DNA content in SNB19 cells 24 and 48 h after IR correlated with the cleavage of PARP, as illustrated in Fig. 7c. The incubation with AZD6244 and/or NVP-BEZ235 did not result in any changes of PARP expression or its cleavage 24 h after IR. However, 48 h after IR a slight increase in cleaved PARP was observed in cells treated with both inhibitors simultaneously, indicating a synergistic cytotoxic effect. In addition, we observed the cleavage of PARP when cells were exposed to 8 Gy, which reflects the enlarged fraction of hypodiploid cells after IR on the protein level.

In contrast to the SNB19 cell line, A549 cells showed significant changes in the hypodiploid fraction after treatment with AZD6244 and NVP-BEZ235. NVP-BEZ235 induced the reduction of the hypodiploid fraction 24 h after IR in A549 cells (Fig. 7b). Furthermore, 48 h after IR an increased proportion of hypodiploid cells was detected after incubation with AZD6244. This increase was further enhanced, when A549 cells were treated with both inhibitors simultaneously. The elevated proportion of hypodiploid cells in AZD6244 treated samples was also observed in irradiated A549 cells. The incubation with NVP-BEZ235 also resulted in a significantly increased proportion of irradiated A549 cells with hypodiploid DNA content. Most strikingly and as already observed for unirradiated samples, the combination of both inhibitors caused the highest fraction of hypodiploid A549 cells 48 h after IR in irradiated cells.

The simultaneous incubation with both inhibitors caused the highest levels of cleaved PARP in unirradiated and irradiated A549 cells 24 h after IR pointing to the induction of apoptosis. Interestingly, 48 h after IR elevated cleaved PARP levels were detected in A549 cells treated with AZD6244, NVP-BEZ235 and a combination of both inhibitors, whereas an elevated proportion of hypodiploid cells was not observed for NVP-BEZ235 treated cells. However, as shown in the flow cytometric data, the combination of both inhibitors resulted in strongest effects in unirradiated A549 cells. Interestingly, in irradiated A549 cells treatment with NVP-BEZ235 alone caused a higher level of PARP cleavage then incubation with both inhibitors 48 h after IR.

To assess the impact of AZD6244, NVP-BEZ235 and IR on the induction of autophagy, we probed for the autophagy marker protein LC3, which is converted from the cytosolic soluble LC3-I to the membrane-bound LC3-II form during the autophagic process. As shown in Fig. 7c, treatment of SNB19 cells with AZD6244, NVP-BEZ235 and IR had only minor effects on the expression levels of LC3-I and LC3-II. However, in A549 cells the depletion of LC3-I was observed 24 after IR, when the cells were treated with the dual PI3K/mTOR inhibitor. This effect also persisted 48 h after IR and was independent of IR or incubation with AZD6244.

## Discussion

Oncogenic signaling cascades have been identified as potential molecular targets for the treatment of different tumor entities [2, 11, 12, 18, 28, 36, 61]. However, depending on the mutational background of the cancer cell the inhibition of pathways can induce the activation of complementary signaling cascades [31, 34, 35, 42]. A strategy to circumvent these cross activations is the simultaneous inhibition of the other complementary signaling cascades. Especially the simultaneous inhibition of the MAPK and the PI3K/mTOR pathways, which are mutated in a multiplicity of human cancers, yielded promising results in various *in vitro* and *in vivo* studies [3, 14, 23, 46, 54, 64], since these signaling cascades are known to influence proliferation, cell growth, survival and resistance to chemotherapeutics and IR [13, 50, 60].

Although, there is evidence that simultaneous treatment with the MEK inhibitor AZD6244 and the PI3K/mTOR inhibitor NVP-BEZ235 causes synergistic effects on tumor cell proliferation and induction of apoptosis [24, 26, 53, 56, 59], little is known about the radiation response of tumor cells after simultaneous inhibition of the MAPK and the PI3K/mTOR pathways. Therefore, this study was designed to integrate the network signaling and phenotypic data of the radiation response after simultaneous MEK and PI3K/mTOR inhibition in SNB19 and A549 cells, which differ in their mutational status of the MAPK and PI3K/mTOR signaling cascade [30].

As shown in Fig. 1, incubation with AZD6244 or NVP-BEZ235 alone resulted in a dose dependent reduction of the proliferation in SNB19 and A549, although effects of NVP-BEZ235 were of greater magnitude in A549 cells. Since there are no known mutations in the PI3K/mTOR signaling cascade in A549 cells [30], it is likely, that the different tumor entity is the main reason for the different sensitivity towards dual PI3K/mTOR inhibition. Combining NVP-BEZ235 with AZD6244 basically resulted in the same proliferation rates as incubation with NVP-BEZ235, implicating that NVP-BEZ235 is the more effective inhibitor.

To elucidate possible reasons for the observed anti-proliferative effects, we analyzed the expression levels of certain key players of the MAPK and PI3K/mTOR signaling cascades after pathway perturbation with AZD6244 and NVP-BEZ235. As expected and in accordance with published results for different tumor entities, the treatment with AZD6244 and NVP-BEZ235 alone resulted in inhibition of the MAPK and PI3K/mTOR signaling cascade, respectively, as confirmed by reduced expression of p-Erk, p-Akt, p-S6 and p-4E-BP1 (Fig. 2). However, we also observed the induction of feedback loops by the two inhibitors, indicated by elevated levels of MEK1/2 (after MEK inhibition) and Akt phosphorylation (after PI3K/mTOR inhibition) after prolonged incubation with the inhibitors, which has already been reported for NVP-BEZ235 in other glioblastoma cell lines [38, 41, 44]. Noteworthy, extended incubation with NVP-BEZ235 also resulted in reduced Raf-1 expression in both cell lines (Fig. 2a), indicating a crosstalk between the two signaling cascades, as depicted in our putative signaling diagram (Fig. 3) and as already published for other cell lines [3, 23, 46].

Apart from the effects on proliferation and the signaling cascades we also assessed the clonogenic ability of SNB19 and A549 cells after IR and incubation with the inhibitors (Fig. 4). In both cell lines AZD6244 caused a radiosensitization, as reported in other studies [9, 10, 62], although only to a moderate extent. A treatment with NVP-BEZ235 resulted in a more profound radiosensitization in both cell lines, indicating that NVP-BEZ235 is the drug which yielded the greater cytotoxic effects, when combined with IR. The NVP-BEZ235 mediated radiosensitization also is in accordance with published data [20, 21, 51, 73]. However, the fact, that in both cell lines combining AZD6244 and NVP-BEZ235 yielded the same result as the dual PI3K/mTOR inhibitor alone, implies that no synergistic or additive effects occurred in SNB19 and A549 cells in terms of radiosensitivity. This is in contrast to previous published results of combining MAPK and PI3K/mTOR pathway inhibitors using a MEK and an Akt inhibitor in pancreatic cancer cells [67], which again confirms that the results of pathway perturbations highly rely on the inhibitors and the genetic background of the treated cells.

To further assess the phenotypic effects in the two tested cell lines after signaling cascade inhibition, we analyzed the cell cycle phase distributions. The incubation with AZD6244 or NVP-BEZ235 caused cell cycle arrests in the G1-phase in both cell lines, as shown in Fig. 5 and Additional file 3. The combination of both inhibitors resulted in an even more profound arrest in the G1-phase in SNB19 cells, whereas no additive or synergistic effects were observed in A549 cells, which is most likely due to the extensive cytostatic effect of NVP-BEZ235 in this cell line. Our Western blot data of the cell cycle related proteins further confirm the flow cytometric data (compare Fig. 6). A treatment with both inhibitors simultaneously resulted in greatest reduction of p-Rb in SNB19 cells, which is an indicator for a blockade at the G1/S-transition checkpoint [33, 45, 65], whereas in A549 cells treatment with NVP-BEZ235 alone already reduced p-Rb expression levels to a maximum. This enhanced cell cycle arrest in the SNB19 cell line after combined MEK and PI3K/mTOR inhibition, indicates an additive or synergistic anti-proliferative effect of AZD6244 and NVP-BEZ235, which has not been shown for glioblastoma cell lines yet, indicating a therapeutically relevant potential of combining these two inhibitors in this tumor entity.

Combining IR and AZD6244 increased the proportion of G1-phase cells in both cell lines, whereas the combination of NVP-BEZ235 and IR resulted in elevated levels of G2/M-phase cells, as shown previously for other cell lines [37, 38]. A simultaneous inhibition of the MAPK and the PI3K/mTOR signaling cascades in irradiated cells resulted in mixed phenotypic effects as observed for the combination of IR with each inhibitor alone. Apparently the two inhibitors are somewhat counteracting each other, which might be a reason for the lack of synergy in terms of radiosensitization.

To further elucidate the effects of MEK and PI3K/mTOR inhibition, we assessed the induction of apoptosis and autophagy. As shown in Fig. 7, incubation with AZD6244 had no relevant effects on apoptosis and autophagy in SNB19 cells, whereas treatment with NVP-BEZ235 caused a slight induction of autophagy 24 h after IR. Combining both inhibitors increased cleaved PARP levels slightly, indicating apoptosis and validating a synergistic effect of the two drugs 48 h after IR in the glioblastoma cell line. However, IR of SNB19 cells increased the hypodiploid fraction and the expression level of cleaved PARP to a greater extent. Most strikingly, this radiation induced apoptosis was slightly enhanced, when irradiated cells were treated with both inhibitors simultaneously.

This is in contrast to the A549 cell line, indicating a cell line specific effect: Although the highest levels of cleaved PARP in unirradiated cells were observed, when cells were treated with both inhibitors, this was not true for irradiated A549 cells. When A549 cells were irradiated interestingly the highest rate of cleaved PARP was detected in cells treated with NVP-BEZ235 solely. Simultaneous treatment of irradiated cells with AZD6244 and NVP-BEZ235 resulted in lower levels of cleaved PARP (Fig. 7), indicating less induction of apoptosis.

Another difference between the cell lines in their response to AZD6244 and NVP-BEZ235 was observed in autophagy. When SNB19 cells were treated with NVP-BEZ235 only a slight induction of autophagy was observed 24 h after IR, as validated by a slight increase of LC3-II. However, A549 cells revealed a complete depletion of LC3-I but no increase of LC3-II. This can be due to the fact, that within the autophagic process the initially increased LC3-II levels are degraded within a few hours after induction of autophagy [48]. This might indicate that the autophagic process is much faster in the lung carcinoma cell line. The induction of autophagy by NVP-BEZ235 was already reported for breast cancer cells lines and is of utmost interest [37], since the role of autophagy in cancer is currently highly discussed [29, 39, 57]. Furthermore, several research groups demonstrated, that inhibitors of the autophagic flux, such as bafilomycin A or chloroquine, can sensitize cancer cells to IR [4, 72]. Therefore, this NVP-BEZ235-mediated induction of autophagy might be exploited to further enhance the radiosensitization with autophagy inhibitors.

## Conclusions

In this study we clearly demonstrated, that our novel approach of combining AZD6244 and NVP-BEZ235 with IR resulted in pathway perturbations and cell line specific effects. In SNB19 cells mainly synergistic cytostatic effects were observed after simultaneous treatment with AZD6244 and NVP-BEZ235, whereas the combination of the same drugs induced apoptosis to a much greater extent in A549 cells. However, apart from the synergistic effects in unirradiated cells, our study also clearly shows, that there are no additive effects in terms of radiosensitivity, when the two radiosensitizers are used in combination.

One major question, which arises from the data presented in this study, is, if the different effects of the combined treatment with AZD6244 and NVP-BEZ235 in the glioblastoma SNB19 and lung carcinoma A549 cells rely on the different mutational background (SNB19 cells are *TP53* and *PTEN* mutated, whereas A549 cells are *KRAS* mutated) or on the different tumor entity. A systematic approach addressing this question will be the subject of forthcoming *in vitro* and *in vivo* experiments, improving our knowledge about how specific mutations and the cancer cell origin affect the impact of inhibiting the MAPK and the PI3K/mTOR pathways. The data generated in these forthcoming experiments will help to improve our putative signaling network, which we presented in this study, ultimately facilitating valid predictions of how the effects of an inhibitor depend on certain mutations and/or the tumor cell origin. This will pave the way for a personalized therapy regimen, which is based on the genetic background of an individual cancer, in order to enhance therapy outcome.

## Additional files

Additional file 1: Antibodies. List of primary antibodies used in this study. (DOCX 12 kb) Additional file 2: Colony forming assay data. Colony forming efficiencies and radiosensitivity parameters of tumor cells treated with AZD6244 and NVP-BEZ235 derived with the linear quadratic model. (PDF 128 kb) Additional file 3: Representative cell cycle histograms. Representative DNA histograms of SNB19 and A549 cells treated with AZD6244, NVP-BEZ235, a combination of both drugs and IR with 8 Gy. Cells were fixed 30 min, 24 or 48 h after IR, permeabilized, treated with RNase A, stained with PI and analyzed for DNA content. DNA histograms were deconvoluted with the ModFit Software. (PDF 3250 kb)

## Abbreviations

CDK1: Cycline dependent kinase 1
CDK4: Cycline dependent kinase 4
CGM: Complete growth medium
D10: Dose yielding 10 % survival
DMSO: Dimethylsulfoxide
DNA: Desoxyribonucleic acid
ERK: Extracellular signal-regulated kinase
IR: Irradiation
MAPK: Mitogen-activated protein kinase
MEK: Mitogen-activated protein*/*extracellular signal-regulated kinase kinase
mTOR: Mammalian target of Rapamycin
p-Rb: Phospho-Retinoblastomaprotein
PARP: Poly ADP ribose polymerase
PI3K: Phosphoinositide 3-kinase
PI: Propidium iodide
SF2: Surviving fraction at 2 Gy
