# Supplementary material for: Simultaneous perturbation of the MAPK and the PI3K/mTOR pathways does not lead to increased radiosensitization
Source: Radiat Oncol. 2015 Oct 24;10:214. doi: 10.1186/s13014-015-0514-5 (PMC4619315; doi:10.1186/s13014-015-0514-5)
Supplement: Additional file 1: — Antibodies. List of primary antibodies used in this study. (DOCX 12 kb) [file 13014_2015_514_MOESM1_ESM.docx]

The primary antibodies used were rabbit polyclonal anti-PI3K p110, rabbit polyclonal anti-PI3K p85, mouse monoclonal anti–phospho-AKT (Ser 473), rabbit polyclonal anti-AKT, rabbit monoclonal anti–phospho-mTOR (Ser 2448), rabbit polyclonal anti-mTOR, rabbit polyclonal anti–phospho-S6 (Ser 240/244), mouse monoclonal anti-S6, mouse monoclonal anti–phospho-4E-BP1 (Thr37/46), rabbit polyclonal anti-LC3B, rabbit polyclonal anti-PARP (all from Cell Signaling Technology, Danvers, MA), mouse monoclonal anti–β-actin (Sigma, Deisenhofen, Germany) and mouse monoclonal anti–phospho-histone H2AX (Ser 139) fluorescein isothiocyanate conjugate (Millipore, Schwalbach, Germany). Secondary species-specific antibodies for Western blot were labeled with HRP (DAKO, Hamburg, Germany).
