# Supplementary material for: Simultaneous perturbation of the MAPK and the PI3K/mTOR pathways does not lead to increased radiosensitization
Source: Radiat Oncol. 2015 Oct 24;10:214. doi: 10.1186/s13014-015-0514-5 (PMC4619315; doi:10.1186/s13014-015-0514-5)
Supplement: Additional file 2: — Colony forming assay data. Colony forming efficiencies and radiosensitivity parameters of tumor cells treated with AZD6244 and NVP-BEZ235 derived with the linear quadratic model. (PDF 128 kb) [file 13014_2015_514_MOESM2_ESM.pdf]

Cloning efficiencies and radiosensitivity parameters[a] of tumor cells treated with AZD6244 and/or NVP-BEZ235.

Cell line	Treatment	PE (%)	SF2	D10 (Gy)	IF SF2	IF D10
**SNB19**	*Control*	53.7 ± 9	0.81 ± 0.10	9.84 ± 0.89	1.00	1.00
	AZD6244	57.2 ± 11	0.64 ± 0.06	8.24 ± 0.48	1.27	1.20
	NVP-BEZ235	53.2 ± 10	0.62 ± 0.07	7.38 ± 0.39	1.31	1.33
	AZD6244 + NVP-BEZ235	51.8 ± 5	0.63 ± 0.07	7.86 ± 0.88	1.27	1.25
**A549**	*Control*	73.2 ± 7	0.80 ± 0.05	9.81 ± 0.68	1.00	1.00
	AZD6244	78.9 ± 12	0.66 ± 0.05	8.87 ± 0.58	1.21	1.11
	NVP-BEZ235	72.3 ± 9	0.66 ± 0.07	7.15 ± 0.87	1.21	1.37
	AZD6244 + NVP-BEZ235	73.7 ± 10	0.65 ± 0.07	7.06 ± 0.60	1.23	1.39

Abbreviations: [a] Means (± SD) from at least four independent experiments; PE – Plating efficiency at 0 Gy; SF2 – Surviving fraction at 2 Gy; D10 – Radiation dose required to yield 10% survival; IF SF2 – Inhibitory factor for SF2 = SF2 Control/SF2 Inhibitor; IF D10 – Inhibitory factor for D10 = D10 Control/D10 Inhibitor
