# Supplementary figures and images for: Simultaneous perturbation of the MAPK and the PI3K/mTOR pathways does not lead to increased radiosensitization
Source: Radiat Oncol. 2015 Oct 24;10:214. doi: 10.1186/s13014-015-0514-5 (PMC4619315; doi:10.1186/s13014-015-0514-5)

# A SNB19

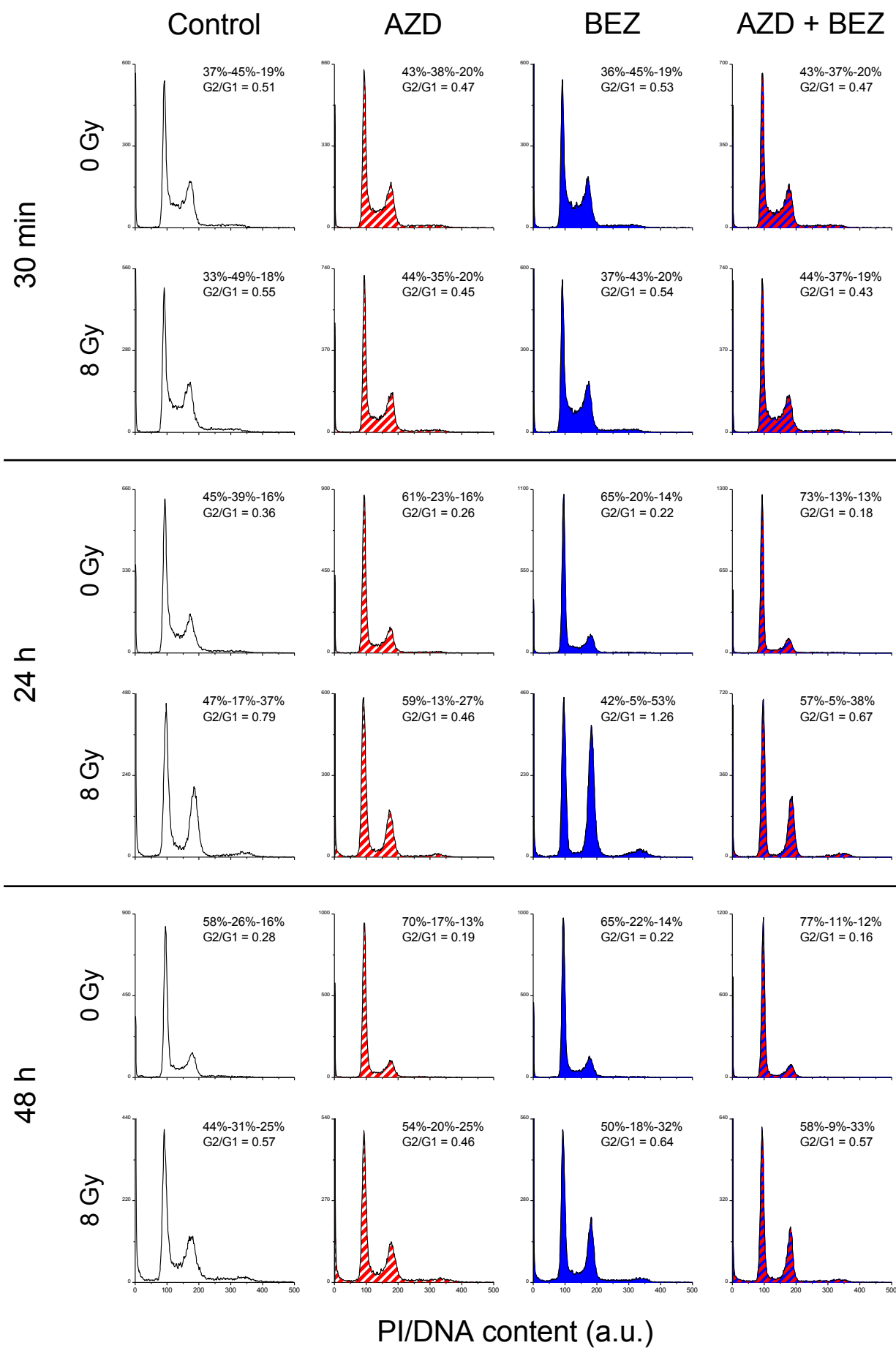

B A549

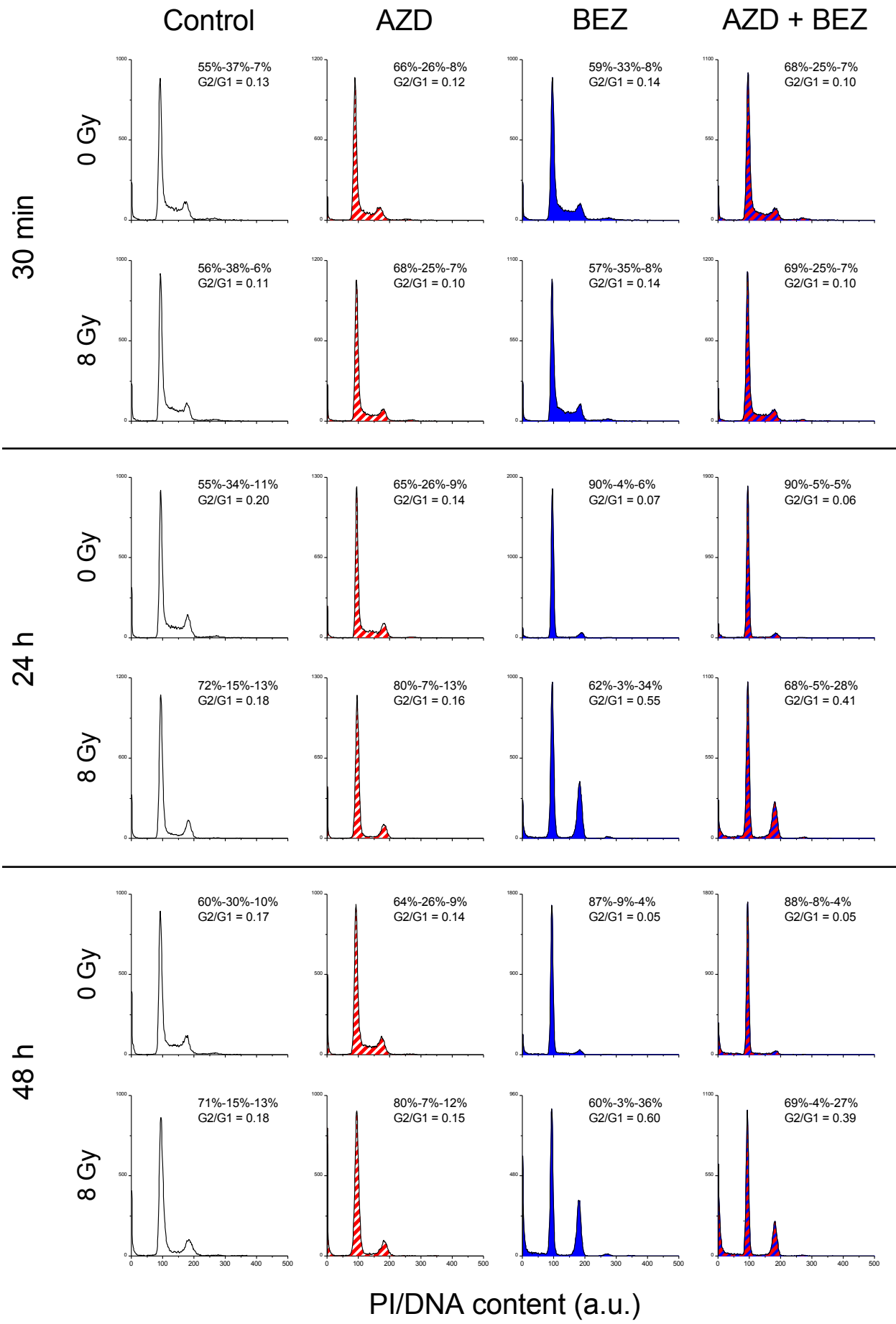

Supplement: Additional file 3: — Representative cell cycle histograms. Representative DNA histograms of SNB19 and A549 cells treated with AZD6244, NVP-BEZ235, a combination of both drugs and IR with 8 Gy. Cells were fixed 30 min, 24 or 48 h after IR, permeabilized, treated with RNase A, stained with PI and analyzed for DNA content. DNA histograms were deconvoluted with the ModFit Software. (PDF 3250 kb) [file 13014_2015_514_MOESM3_ESM.pdf]
